# Hemoptysis as a rare manifestation of missed blunt thoracic aorta injury, a case report

**DOI:** 10.1016/j.ijscr.2023.108918

**Published:** 2023-10-05

**Authors:** Roozbeh Cheraghali, Pezhman Kharazm, Reza Afghani, Dayan Amanian, Navid Hajihoseini

**Affiliations:** Clinical Research Development Center, 5 Azar Hospital, Golestan University of Medical Sciences, Gorgan, Iran

**Keywords:** Blunt thoracic aortic trauma, Hemoptysis, Pseudoaneurysm, Case report

## Abstract

**Introduction and importance:**

Blunt thoracic aorta injury is one of the most fatal injuries in multiple trauma patients and most of these injuries lead to death at the scene. Some patients remain undiagnosed because of the lack of specific symptoms for these injuries. Hemoptysis as a presentation of a neglected blunt aortic trauma is a very rare condition. In this study, we present a case with a 7-month delay in presentation and diagnosis.

**Case presentation:**

A 49-year-old man with a complaint of intermittent hemoptysis was presented to the clinic. He had a history of chest trauma following falling 7 months ago. His physical examination was unremarkable. On Computed Tomography Angiography (CTA) pseudoaneurysm of the descending aorta was detected and the patient was treated urgently with a stent graft.

**Clinical discussion:**

Blunt thoracic aorta injury may occur following deceleration traumas. Descending aorta is the most involved segment but other segments may be involved as well. Bleeding can be stopped by tamponading the aorta with its overlying pleura. In some cases, pseudoaneurysms are formed and may remain undiagnosed for a long time after index trauma. CTA is the most useful diagnostic study and when the diagnosis is made, urgent treatment is mandatory. Although endovascular repair has significantly lower mortality and morbidity, open surgical repair may be inevitable in some cases.

**Conclusion:**

Thoracic aorta injury should be suspected in any patient with severe deceleration trauma and CTA should be used promptly for the diagnosis and treatment of these potentially fatal injuries.

## Highlights

• Blunt thoracic aorta injury is the second cause of mortality in trauma patients.
• Diagnosis of blunt aortic injury in asymptomatic patients needs a high grade of clinical competence.
• CT angiography should be performed for any case whose trauma mechanism is suspicious for blunt thoracic aorta injury.
• Some of the undiagnosed blunt aortic injury patients may become symptomatic several months after initial trauma.
• Although very rare, hemoptysis may be the result of an aortic pseudoaneurysm eroding the bronchial wall.
• Endovascular repair has a lower mortality and morbidity in the treatment of blunt thoracic aorta injury.

## 1 Introduction

Blunt Thoracic Aortic Injury (BTAI) is one of the most fatal conditions trauma victims may meet [1]. It is the second cause of mortality in blunt trauma patients, after closed head injury [2]. More than 80 % of patients die at the scene and without prompt diagnosis and treatment, significant morbidity and even mortality threaten those patients who arrive in hospital alive [3]. Descending thoracic aorta just distal to the subclavian artery is the most common segment involved [4]. Diagnosis of BTAI requires a high grade of clinical suspicion and performing prompt radiologic studies [5]. There are few reports of delayed diagnosis of thoracic aortic injury and hemoptysis is a very rare manifestation of BTAI [[6], [7], [8]]. In this study, we present a case of BTAI presented with intermittent hemoptysis 7 months after a falling.

The study has been reported in line with the SCARE criteria [9].

## 2 Case report

**Fig. 1:**
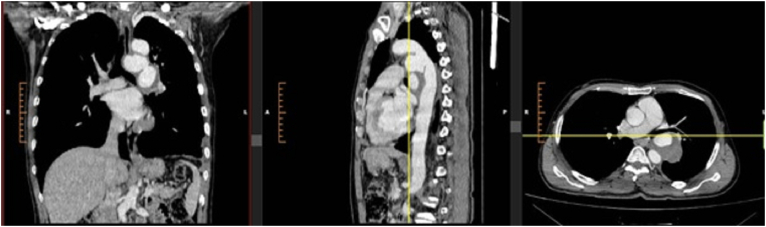
Coronal, sagittal, and axial computed tomography angiography (CTA) demonstrating bilobed pseudoaneurysm of the descending aorta with pressure effects on the distal trachea and left main bronchus.

A 49-year-old man came to the clinic because of episodes of hemoptysis. He had no accompanying symptoms including chronic cough, sweating, or weight loss. Episodes were not related to digestion or any other condition and contained little fresh blood. He denied any history of significant pulmonary infection including tuberculosis. He had a history of falling from a height of 6 m 7 months ago causing left-sided hemothorax and was treated with a tube thoracostomy. No other information was available about that incident. On physical examination, he had normal vital signs. Chest examination revealed no significant finding except for the scar of tube thoracostomy. Other organs were unremarkable. He underwent a thoracic CT angiography. On CT angiogram descending aortic injury distal to the left subclavian artery branch of the aortic arc was seen. There were at least two contrast-enhanced outpouchings with diameters of about 50 mm on the medial and lateral sides of the descending aorta containing thrombosis. One of them had close proximity to the left main bronchus. (Fig. 1).

**Fig. 2:**
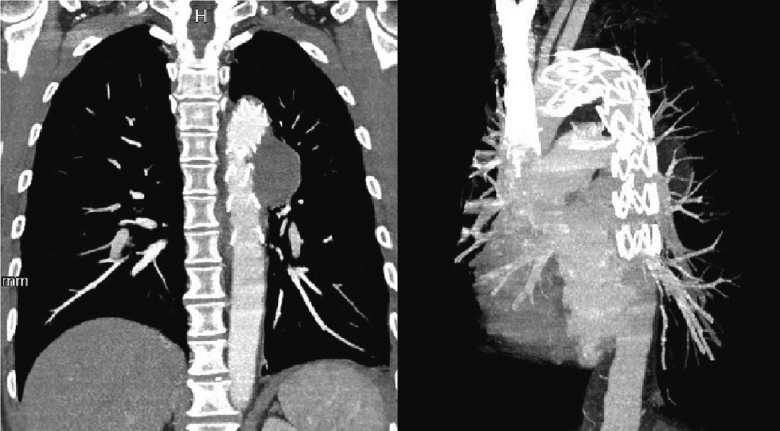
Coronal and three-dimensional computed tomography angiography (CTA) shows stent graft in the descending aorta as well as complete thrombosis of pseudoaneurysm.

As soon as the CTA was reviewed, the patient was scheduled for endovascular repair of the thoracic aorta. In a hybrid operation room and under general anesthesia, vascular access was obtained from right common femoral artery. Digital subtraction angiography was performed and critical points were marked, including the left subclavian artery orifice, proximal margin and distal extension of injury, and the diameter of the aorta (Video 1). After final measurements, a 34 ∗ 161 stent graft was deployed in the aorta from the inferior margin of the left subclavian artery to the distal of the injured segment of the aorta, excluding pseudoaneurysms from the circulation. Completion angiography showed no contrast blush in regular or delayed sequences (Video 2). The patient left the hospital 2 days after the operation and hemoptysis did not occurred then after. CT angiography 2 months later revealed remodeling of the thoracic aorta and complete thrombosis of pseudoaneurysms (Fig. 2).

## 3 Discussion

Blunt thoracic aortic injury is one of the most lethal conditions in multiple trauma patients [1]. Rapid deceleration following falling from a significant height or high speed motor vehicle collisions are the most common mechanisms of this trauma [10].

This injury is classified based on the extent of the damage through the arterial wall.

Grade I includes injuries limited to the intima. Grade II injuries refer to intramural hematomas. In grade III (as in our case), all of the layers of the arterial wall are destructed but a thin layer of adventitia or parietal pleura prevents free rupture into the pleural cavity, thus creating a contained outpouching entitled “Pseudoaneurysm”. At last, grade IV refers to free rupture of the artery [11].

Our patient had a history of falling several months ago and it was the most relevant cause that could be attributed as the cause of aortic injury.

Diagnosis of thoracic aortic injury requires a high degree of clinical suspicion. Most of patients don't have specific findings on physical examination, and routine radiologic studies can't reliably rule out these injuries [2]. When the mechanism of trauma raises suspicion, advanced radiologic evaluation is mandatory. CT angiography with a sensitivity and specificity of more than 95 % is the imaging study of choice when a thoracic aortic injury is probable [5]. Our case had a history of admission following trauma and a thoracostomy tube. The aortic injury was not diagnosed at that time.

When a traumatic aortic injury is diagnosed in the acute phase of trauma, all patients should be admitted to the intensive care unit (ICU) for monitoring and control of blood pressure and pulse rate to reduce the stress on the aortic wall. In patients whose diagnosis is delayed for any reason, as in our subject, appropriate treatment should be given as soon as possible based on the degree of injury [12].

Most of the grade I injuries are treated with this conservative management. Grade III and IV injuries require intervention that can be open or endovascular. Endovascular intervention is preferred considering its lower morbidity and mortality. In grade II of injuries, some guidelines recommend intervention, but most of studies indicate that conservative management is safe and effective [[12], [13], [14], [15], [16]]. Hemoptysis as the late presentation of BAO is a very rare scenario and when diagnosed, should be treated emergently otherwise, the patient may suffocate following frank bleeding to the bronchi [7].

## 4 Conclusion

When the trauma is severe, thoracic aortic injury should be suspected as a potential complication that can be disastrous. CT angiography can reliably diagnose and grade the injury and when intervention is necessary, treatment should be applied as soon as possible.

Finally, it should be emphasized that detailed history taking and physical examination is the key point of any successful management of challenging cases. In this case, if the patient's history of trauma was ignored, the patient would not be so lucky.

The patient's full consent was obtained for the publication of this article and images.

The following are the supplementary data related to this article.

Video 1 Digital Subtraction Angiography indicating pseudoaneurysm in descending aorta. Video 1

Video 2 Completion angiography reveals exclusion of pseudoaneurysm by stent graft. Video 2

## Ethical approval

This study was approved by the Golestan University of Medical Sciences Research Ethics Committee with the following ethics code: https://ethics.research.ac.ir/IR.GOUMS.REC.1402.221.

Date of approval: 12/Sep/2023.

## Funding

There is no funding source for this study.

## Author contribution

Dr. Roozbeh Cheraghali, vascular surgeon, endovascular interventionist of the patient.

Dr. Pezhman Kharazm, vascular surgeon and the patient's corresponding physician.

Dr. Dayan Amanian, radiologic consultant.

Dr. Reza Afghani, thoracic surgery consultant.

Dr. Navid Hajihoseini, assistant surgeon and literature reviewer.

## Guarantor

Dr. Pezhman Kharazm

## Declaration of competing interest

There is no conflict of interest between authors.
